# Looking for atomic precision in nanochemistry

**DOI:** 10.1038/s42004-023-01057-3

**Published:** 2024-02-05

**Authors:** Rodolphe Antoine, Qiaofeng Yao, Kui Yu

**Affiliations:** 1 Institut Lumière Matière UMR 5306, Université Claude Bernard Lyon 1, CNRS, Univ Lyon, F69100 Villeurbanne, France; 2 Tianjin Key Laboratory of Molecular Optoelectronic Sciences, Key Laboratory of Organic Integrated Circuits, Ministry of Education, Department of Chemistry, School of Science, Tianjin University, Tianjin, 300072 P. R. China; 3 Engineering Research Center in Biomaterials, Sichuan University, Chengdu, 610065 P. R. China

## Abstract

The Guest Editors of *Communications Chemistry*’s Atomically precise nanochemistry Collection discuss the importance of atomic precision in nanochemistry research, and highlight some of the field’s most pressing challenges.

Nanomaterials produced by various approaches usually display dispersity in their shapes, sizes, and compositions, which play a significant role in determining their physicochemical properties. Synthesizing nanomaterials with atomic precision is therefore of importance. With well-defined structure–property relationships, it is possible to achieve reliable and programmable performances in a diverse range of applications of interest. The need for atomic precision becomes imperative when the characteristic size of the nanomaterials decreases to a few nanometers or even the sub-nanometer scale. In this size range similar to molecules, every atom counts. Upon configurational changes, or addition or removal of a single atom, properties may vary significantly and in unpredictable ways. Properties are affected by the spatial distribution of elements and by nanomaterial size. Because every atom counts towards defining properties, it is critically important to develop robust synthetic approaches, such that “nanochemistry” may efficiently manufacture nanoscale materials with atomic precision.

Recent efforts in nanochemistry have allowed the synthesis of a range of atomically precise nanomaterials, including metal nanoclusters, semi-conductor magic-size clusters, polyoxometalate clusters, carbon-based nanostructures, and inorganic nanocrystals. These nanomaterials achieve a level of structural precision similar to that of molecules.

Ligand-protected metal nanoclusters of atomic precision are the class of nanomaterial that this Collection features most prominently. The Brust-Schiffrin method introduced in 1994 is the seminal cornerstone for atomically precise nanochemistry, which uses a two-phase method to produce narrow-sized nanoparticles^1^. The wet-chemical synthesis addressed in this Collection and elsewhere now uses not only thiolates as protecting ligands, but also other organic molecules, such as alkynyls, N-heterocyclic carbenes, and multidentate nitrogen donor ligands. Alongside wet chemistry, gas-phase routes such as vapor deposition techniques are also capable of producing atomically precise clusters.

We are, however, far from being able to consistently synthesize all nanomaterials with an atomically precise level of control, and a fundamental understanding of the precise growth mechanisms is missing in general. Such understanding will allow us to synthesize nanomaterials in such a way that their composition, size, shape, and other property-dictating attributes are precisely controlled. This is particularly true for wet-chemistry synthesis, which typically involves numerous chemicals in a single reaction. Even a small change in one of the experimental parameters can have subtle and difficult-to-quantify effects on the reaction products.

For nanomaterial assemblies, smart linkers that fuse building blocks together in a precise and predictable manner are in demand. Such assemblies allow for the properties of different materials to be combined, and enable access to new, even synergetically combined functions. Expertise from supramolecular chemists will be of help in tackling this challenge.

In addition, computational and theoretical chemistry are needed to advance our understanding of structure–property correlations, together with the dynamic processes involved in nanomaterial formation. Advances in experimental characterization tools are also imperative, with high-resolution mass spectrometry being one of the emerging techniques available for metal nanoclusters.

This Collection presents some of the latest progress in the synthesis, functionalization, characterization, self-assembly, and application of atomically precise nanomaterials. In the synthesis section we showcase routes together with the factors and principles governing nanomaterial formation. In the section that follows, recent advances in the characterization, modulation, conversion, and hybridization of pre-formed nanomaterials at atomic precision are presented. Finally, we showcase research aiming to establish structure–property relationships at atomic precision.

We would like to extend our congratulations to the three winners of the Nobel Prize in Chemistry 2023 “for the discovery and synthesis of quantum dots”. Quantum dots represent a seminal development for nanotechnology. We hope that the cutting-edge research advances in the three aforementioned sections provide the community with a useful reference for achieving atomic precision in nanotechnology, stimulating more interest on advancing the precision of nanotechnology in the near future.
